# Evaluation of biometry and corneal astigmatism in cataract surgery patients from Central China

**DOI:** 10.1186/s12886-017-0450-2

**Published:** 2017-04-26

**Authors:** Ji-guo Yu, Jie Zhong, Zhong-ming Mei, Fang Zhao, Na Tao, Yi Xiang

**Affiliations:** Department of Ophthalmology, the Central Hospital of Wuhan, Tongji Medical College, Huazhong University of Science and Technology, No, 26 Shengli Street, Wuhan, Hubei Province 430014 China

**Keywords:** Biometry, Corneal astigmatism, Cataract, IOLMaster device, Central China

## Abstract

**Background:**

To evaluate the distribution of biometric parameters and corneal astigmatism using the IOLMaster device before phacoemulsification in cataract patients in Central China.

**Methods:**

Consecutive cataract patients were recruited at the Central Hospital of Wuhan between January 2015 and June 2016. Ocular axial length (AL), keratometry values, anterior chamber depth (ACD) and horizontal corneal diameter (white to white [WTW]) of each cataract-affected eye were measured with the IOLMaster device.

**Results:**

The study evaluated 3209 eyes of 2821 cataract patients. The mean AL, ACD, and WTW were 24.38 ± 2.47 mm, 3.15 ± 0.48 mm, and 11.63 ± 0.43 mm, respectively. Corneal astigmatism of 0.51–1.00 diopters (D) was the most common range of values (34.96%). A total of 10.56% patients exhibited a corneal astigmatism greater than 2.0 D. The flat and steep keratometry values gradually increased with age. The mean ACD and WTW showed increasing trends as the AL increased (*P* < 0.001). When the AL was shorter than 26.0 mm, the keratometry decreased as AL increased. The against-the-rule (ATR) astigmatism proportion increased with age and the with-the-rule (WTR) astigmatism proportion decreased with age.

**Conclusions:**

The profile of ocular biometric data and corneal astigmatism may help ophthalmologists improve their surgical procedures and make an appropriate IOL choice to gain a high quality of postoperative vision.

## Background

Cataract is the leading cause of blindness and the only form of treatment is surgery. Phacoemulsification is the most commonly used and effective surgical method for the treatment of cataract worldwide. Accurate measurement of ocular axial length, keratometry, anterior chamber depth and corneal diameter before cataract surgery is crucial for obtaining the precise degree of implanted intraocular lens (IOL) to control the postoperative diopter (D) value plus or minus 0.50 D as well as to achieve satisfactory postoperative refractive results and improve the visual quality for cataract patients [1, 2].

Partial coherence interferometry (IOLMaster, Carl Zeiss Meditec, Germany) is a type of optical coherent biological measuring instrument that utilizes non-contact technology to measure axial length, keratometry, anterior chamber depth and corneal diameter. With its ultra-high precision (5 mm or less) and good resolution (12 mm), it is widely used in evaluating the ocular parameters and IOL calculations in cataract patients before surgery. Through its innovative and accurate measurement of ocular parameters, the degree of intraocular lens can be accurately calculated before implantation [3]. Corneal astigmatism is also a major factor affecting postoperative visual quality. The IOLMaster can measure preoperative corneal astigmatism and predict the residual corneal astigmatism after cataract surgery [4].

However, most previous studies of preoperative ocular biometry and corneal astigmatism on cataract patients focused on the European and American populations [5–8]. Although there have been some domestic related studies, they aimed to evaluate the Southern, Northern, and Eastern Chinese populations [1, 9, 10]. However, the epidemiological investigation of ocular biometry and corneal astigmatism of cataract patients in the Central China region has yet to be investigated. Therefore, the aim of our study was to evaluate the distribution of biometric parameters, and determine the prevalence of corneal astigmatism using the IOLMaster measurement device before phacoemulsification in cataract patients in Central China, to provide some reference for improving cataract surgical procedures and designing an intraocular lens to meet eye characteristics of the Central Chinese population.

## Methods

### Subjects

This study was approved by the institutional ethics committee of the Central Hospital of Wuhan (Hubei, Central China), and followed the tenets of the Declaration of Helsinki. Consecutive cataract patients scheduled for phacoemulsification and foldable IOL implantation were recruited at the Central Hospital of Wuhan between January 2015 and June 2016. All patients who were local residents of Central China, had cataract, and were older than 30 years were included. Exclusion criteria included a history of ocular surgery, such as refractive surgery, corneal diseases, ocular inflammation, and trauma; patients from other areas of China were also excluded. Routine eye examinations were performed before surgery, including visual acuity, refraction, tonometry, slit lamp evaluation, and dilated fundus evaluation. The procedures were fully explained to each patient, and they provided written informed consent.

### Biometry examination

Ocular axial length (AL), keratometry values, anterior chamber depth (ACD) and horizontal corneal diameter (white to white [WTW]) of each cataract-affected eye were measured with the IOLMaster (Carl Zeiss Meditec, Germany, software version 5.4). Keratometry was measured in 2 meridians: that is, flat keratometry (K1) and steep keratometry (K2). The K value was calculated as the mean of K1 and K2. The patients were divided into 7 groups on the basis of age as follows: 30–40 years, 41–50 years, 51–60 years, 61–70 years, 71–80 years, 81–90 years, and 90 years and older. All eyes were stratified into 4 groups based on AL as follows: shorter than 22.0 mm, 22.0–24.5 mm, longer than 24.5 mm–26.0 mm, and longer than 26.0 mm.

### Statistical analysis

All data were recorded in Microsoft Excel spreadsheets, and analyzed using the Kolmogorov-Smirnov test for normal distribution. Continuous variables were expressed as the mean ± standard deviation for those displaying normal distribution. One-way analysis of variance and the Kruskal-Wallis test were applied for the comparison of variance for normally and non-normally distributed data among the different age groups, respectively. Statistical analysis was performed using SPSS PASW Statistics Version 18.0 software (IBM Corporation, Armonk, NY, USA). *P*-values less than 0.05 were considered statistically significant.

## Results

### Distribution of ocular biometry

**Fig. 1:**
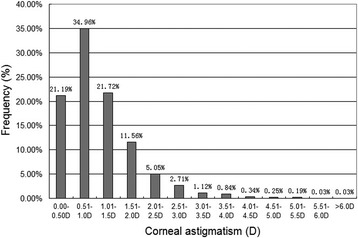
Distribution of corneal astigmatism

This study evaluated 3209 eyes of 2821 cataract patients. The patient demographics are shown in Table 1, which also shows a comparison of these demographics with 4 other published papers that studied populations from the different regions in China. The histograms of the frequency distribution of corneal astigmatism for all patients are shown in Fig. 1. Corneal astigmatism of 0.51–1.00 D was the most common range of values (34.96%), followed by 1.01–1.50 D (21.72%), 0.0–0.50 D (21.19%), and 1.51–2.0 D (11.56%). A total of 10.56% patients exhibited a corneal astigmatism greater than 2.0 D.

**Table 1:** Comparison of demographic features between the present study and 4 other published studies

Parameters	Present	Cui [1]	Chen [11]	Yuan [9]	Guan [10]
Location	Central China	Southern China	Southern China	Northern China	Eastern China
Eyes/patients	3209/2821	6750/4561	4831/2849	12,449/6908	1430/827
Age (y)					
Mean ± SD	70.51 ± 9.81	70.4 ± 10.5	70.56 ± 9.55	69.80 ± 11.15	72.27 ± 11.59
Range	32, 95	40, 101	40, 95	30, 97	16, 98
Male/female	1071/1750	2026/2535	1090/1759	3199/3709	359/468
Keratometry (D)					
Mean ± SD	1.09 ± 0.77	0.90(Median)	1.01 ± 0.69	1.15 ± 0.84	1.07 ± 0.73
Range	0.0, 6.21	NR	0.05, 6.59	0.0, 6.63	0.06, 5.52
K1 mean ± SD	43.75 ± 1.59	43.57 ± 1.69	43.76 ± 1.53	43.93 ± 1.67	43.57 ± 1.56
K2 mean ± SD	44.84 ± 1.65	44.69 ± 1.69	44.76 ± 1.56	45.08 ± 1.73	44.64 ± 1.65
K mean ± SD	44.29 ± 1.58	44.13 ± 1.63	NR	NR	NR
Corneal astigmatism (%)					
≤ 0.5D	21.19%	NR	23.14%	20.76%	21.2%
≥ 1.0D	43.85%	43.9%	41.3%	47.27%	45.46%
≥ 2.0D	10.56%	11.6%	8.22%	13.16%	10.42%
≥ 3.0D	2.80%	3.4%	1.68%	3.75%	2.31%
axis	88.82 ± 49.63	NR	NR	NR	NR
AL (mm)	24.38 ± 2.47	24.07 ± 2.14	23.58 ± 1.13	NR	NR
ACD (mm)	3.15 ± 0.48	3.01 ± 0.57	NR	NR	NR
WTW (mm)	11.63 ± 0.43	11.68 ± 0.45	NR	NR	NR

*D* diopter, *K1* flat keratometry, *K2* steep keratometry, *K* mean keratometry, *SD* standard deviation, *AL* axial length, *ACD* anterior chamber depth, *WTW* white to white, *NR* not reported

### Different age groups

**Fig. 2:**
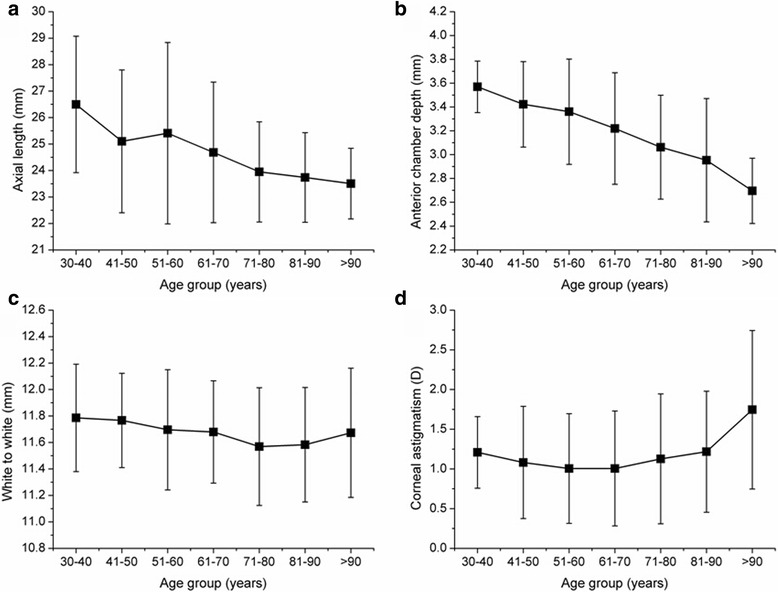
The biometric parameters stratified and analyzed according to age. **a** Axial length, **b** Anterior chamber depth, **c** White to white, **d** Corneal astigmatism

Table 2 shows the mean and standard deviation values of all measured biometric parameter values in the 7 different age groups. The flat and steep keratometry values gradually increased with age. Most eyes in this cohort were between 71 and 80 years old (34.66%), followed by 61 and 70 years old (33.04%). In addition, the AL, ACD, and WTW values showed a gradually decreasing trend with age; corneal astigmatism showed first a decline and then a rising trend (Fig. 2).

**Table 2:** Descriptive statistics for the 7 age groups

Age Group (Y)	AL (mm)	ACD (mm)	WTW (mm)	Astigmatism (D)	K1 (D) mean ± SD	K2 (D) mean ± SD	K (D) mean ± SD	Eyes (%)
30–40	26.50 ± 2.58	3.57 ± 0.22	11.79 ± 0.41	1.21 ± 0.45	42.86 ± 1.52	44.07 ± 1.74	43.47 ± 1.62	13(0.41%)
41–50	25.10 ± 2.70	3.42 ± 0.36	11.77 ± 0.36	1.08 ± 0.71	42.87 ± 1.33	43.95 ± 1.43	43.41 ± 1.33	61(1.90%)
51–60	25.41 ± 3.42	3.36 ± 0.44	11.70 ± 0.45	1.01 ± 0.69	43.74 ± 1.74	44.75 ± 1.76	44.24 ± 1.71	422(13.15%)
61–70	24.68 ± 2.66	3.22 ± 0.47	11.68 ± 0.39	1.01 ± 0.72	43.81 ± 1.56	44.82 ± 1.64	44.32 ± 1.55	1060(33.04%)
71–80	23.95 ± 1.89	3.06 ± 0.44	11.57 ± 0.44	1.13 ± 0.82	43.78 ± 1.56	44.91 ± 1.62	44.35 ± 1.53	1112(34.66%)
81–90	23.74 ± 1.69	2.95 ± 0.52	11.58 ± 0.43	1.22 ± 0.76	43.68 ± 1.60	44.89 ± 1.65	44.29 ± 1.58	514(15.99%)
>90	23.50 ± 1.33	2.70 ± 0.27	11.67 ± 0.49	1.75 ± 1.00	43.67 ± 2.07	45.42 ± 1.93	44.54 ± 1.93	27(0.85%)
*P*-value	< .001	< .001	< .001	< .001	< .001	< .001	< .001	

*Y* years, *mm* millimeter, *D* diopter, *K1* flat keratometry, *K2* steep keratometry, *K* mean keratometry, *SD* standard deviation, *AL* axial length, *ACD* anterior chamber depth, *WTW* white to white

### Distribution of axial length

Table 3 shows the distribution of ocular biometry for different ALs. The AL in the majority of eyes was between 22.0 and 24.5 mm (63.63%). The mean ACD and WTW showed increased as the AL increased (*P* < 0.001). When the AL was shorter than 26.0 mm, the keratometry values (K1, K2, and K) decreased with an increase in AL. However, this trend seemed to revert in patients with an AL of more than 26.0 mm. The smallest mean corneal astigmatism (1.03 D) was in eyes with an AL between 22.0 and 24.5 mm, and the largest (1.26 D) was in eyes with a longer AL than 26.0 mm.

**Table 3:** The distribution of ocular biometry for different ALs

		Mean ± SD					
				Keratometry (D)			
AL (mm)	Eyes, n (%)	ACD (mm)	WTW (mm)	K1	K2	K	Astigmatism (D)
Shorter than 22.0	218 (6.80%)	2.79 ± 0.48	11.38 ± 0.46	45.39 ± 1.40	46.51 ± 1.41	45.95 ± 1.36	1.12 ± 0.75
22.0–24.5	2042 (63.63%)	3.01 ± 0.44	11.62 ± 0.42	43.85 ± 1.41	44.88 ± 1.45	44.37 ± 1.39	1.03 ± 0.72
Longer than 24.5–26.0	390 (12.15%)	3.35 ± 0.37	11.66 ± 0.44	43.00 ± 1.70	44.14 ± 1.97	43.57 ± 1.79	1.15 ± 0.87
Longer than 26.0	559 (17.42%)	3.50 ± 0.40	11.72 ± 0.37	43.32 ± 1.71	44.58 ± 1.78	43.95 ± 1.69	1.26 ± 0.83
Total	3209 (100%)	3.15 ± 0.48	11.63 ± 0.43	43.75 ± 1.59	44.84 ± 1.65	44.29 ± 1.58	1.09 ± 0.77
*P*-value		< .001	< .001	< .001	< .001	< .001	< .001

*mm* millimeter, *D* diopter, *K1* flat keratometry, *K2* steep keratometry, *K* mean keratometry, *SD* standard deviation, *AL* axial length, *ACD* anterior chamber depth, *WTW* white to white

### Distribution of corneal astigmatism

**Fig. 3:**
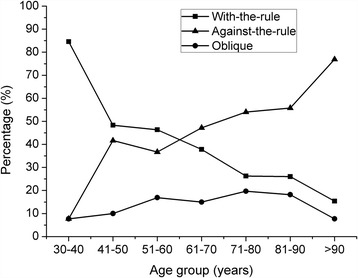
Percentages of with-the-rule, against-the-rule, and oblique corneal astigmatisms in the 7 groups

Corneal astigmatism was with-the-rule (WTR, the steepest meridian of the cornea being within 90 ± 30 degrees) in 1186 eyes (36.96%), against-the-rule (ATR, the steepest meridian of the cornea being within 180 ± 30 degrees) in 1535 eyes (47.83%), and oblique (steepest meridian between 30 and 60 degrees or 120 and 150 degrees) in 488 eyes (15.21%). The ATR astigmatism proportion increased with age and the WTR astigmatism proportion decreased with age. The proportion of oblique astigmatism changed little with increasing age. The percentages of WTR, ATR, and oblique corneal astigmatisms in the 7 groups are shown in Fig. 3.

## Discussion

This study evaluated the distribution of ocular biometric parameters and characteristics of corneal astigmatism measured using the IOLMaster device in cataract patients residing in Central China. Studies of corneal astigmatism in cataract patients from Southern [1, 11], Northern [9] and Eastern [10] China have previously been published. We compared these previous results with those in the present study and found that the corneal power in patients from Northern China was greater than that in other different regions, while the difference among other areas was not obvious. The highest astigmatism in patients from the four areas did not exceed 7.00 D, but the percentage of cataract patients with astigmatism higher than 1.00 D was the greatest in Northern China. This is probably attributed to a regional difference in the populations, as well as different environmental and life style factors [12].

Corneal astigmatism across all age groups showed a similar distribution pattern compared to previous studies [6, 8, 9, 13]. The vast majority of eyes with cataract had a corneal astigmatism between 0.5 D and 1.0 D. In contrast, only a small percentage of eyes with corneal astigmatism greater than 3.0 D were observed. Understanding the distribution of astigmatism is important to help ophthalmologists choose first-line treatment that will be most effective and reduce the occurrence of postoperative astigmatism. This includes procedures such as limbal relaxing incisions [14], opposite clear corneal incisions [15], excimer laser refractive procedures [16, 17], femtosecond laser-assisted astigmatic keratotomy [18], and toric IOL implantation [19–22]. At present, toric IOL implantation is highly recognized and it can be used to correct up to 8.0 D of corneal astigmatism after cataract surgery [23, 24]. In the present study, 21.28% of cataract patients in Central China had corneal astigmatism values between 1.5 D and 4.0 D, most of which could be effectively corrected with toric IOLs. Therefore, the use of toric IOLs in Central China is still required, and that is not less than the demand in other parts of China.

The mean AL, ACD and WTW in the present study are consistent with that reported by Cui et al. [1], who reported on biometry characteristics of the Southern China population. Unfortunately, the data mentioned above were not reported for the Northern and Eastern China populations; therefore, we were unable to make a comparison with those studies. All biometric parameters that were measured using the IOLMaster device were presented as significant differences between age groups. The AL, ACD, and WTW values gradually decreased with age; corneal astigmatism showed an initial decline and then subsequently an increase. This suggests that the human eye biometric parameters change with age. This might be related to the occurrence of lens opacity and thickening, accommodative lags, cornea arcus senilis, extraocular muscle relaxation and orbital fat prolapse generating compression on the eye.

Ocular axial length affects other components of the biometric parameters in eyes. In the present study, we found that as the AL increased, ACD and WTW also increased. Additionally, the keratometry values (K1, K2, and K) decreased when the AL was between 20.0 and 26.0 mm. These results are consistent with the findings reported in previous studies [1, 6, 25, 26]. This suggests that the cornea becomes flatter when the AL increases, accompanied by a larger horizontal WTW. However, this characteristic was not observed when the AL was greater than 26.0 mm. ATR astigmatism accounted for the majority of the cataract population, and the prevalence increased with age. By contrast, the percentage of WTR astigmatism decreased with age. These findings are consistent with the characteristics seen in populations from different countries and regions [6, 9, 27, 28]. These changes have been found to be due to a discrepancy in eyelid morphology and power [29].

It is well known that a toric IOL is indicated when there is a corneal astigmatism of 1.50 D or more. Although the IOLMaster measures six points of the central corneal surface within a 2.3 mm range, it is unable to reflect the entire corneal surface curvature. The pupil is only approximately 2.0–3.0 mm wide during the day, and considering the long duration of daytime eye use in most people, the IOLMaster mainly reflects the results of the central corneal astigmatism; therefore, the IOLMaster measurements also have reference values. Corneal topography can measure the total corneal astigmatism, and is more accurate for distinguishing between regular and irregular astigmatism. Therefore, we believe that for the selection of toric IOLs, one should consider both the corneal topography and IOLMaster measurements in order to make a comprehensive judgment. The current study found that a total of 710 (22.13%) eyes in our study were potential candidates, however when considering implantation of toric IOLs, other factors such as the surgical techniques, economic feasibility for the patient, and rotation of the optical axis should also be taken into account. Total astigmatism is determined by corneal astigmatism, which is the major factor affecting postoperative visual quality; therefore it is crucial to select a reasonable and economical operative procedure to correct corneal astigmatism [1]. The most cost-effective methods to reduce corneal astigmatism are to make smaller incision and choose the most appropriate location for the corneal incision. Our study reported that ATR astigmatism accounted for the majority of the cataract population, and that prevalence increased with age. The characteristics of corneal astigmatisms in our study suggest that when considering large-scale cataract surgery for patients with a low socioeconomic status in Central China, smaller and temporal corneal incisions should be used frequently to reduce preexisting corneal astigmatism, especially in the underdeveloped areas in China.

Our study has some limitations. First, the ocular biometric data drawn from the cataract patients in our hospital do not completely represent the data of the whole population in Central China. Second, we did not make a comparative analysis of eye biometric parameters with data reported abroad because previous studies reported much more detail, and we did not compare findings between men and women. Furthermore, we did not assess the relationship between biometric parameters and genetics, diet, education, occupation, and the severity of the cataract due to lack of relevant data.

## Conclusions

In conclusion, our study determined the distribution of ocular biometric parameters and the characteristics of corneal astigmatism as well as their variation among different age groups in Central China. The profile of ocular biometric data and corneal astigmatism may help ophthalmologists improve their surgical procedures including appropriate IOL choice and more accurate corneal incision made to gain a high quality of postoperative vision.

## Abbreviations

ACD: Anterior chamber depth
AL: Axial length
ATR: Against-the-rule
D: Diopter
IOL: Intraocular lens
K: Mean keratometry
K1: Flat keratometry
K2: Steep keratometry
Mm: Millimeter
NR: Not reported
SD: Standard deviation
WTR: With-the-rule
WTW: White to white
Y: Years
